# Multidrug Resistant and Extensively Drug Resistant Bacteria: A Study

**DOI:** 10.1155/2016/4065603

**Published:** 2016-01-28

**Authors:** Silpi Basak, Priyanka Singh, Monali Rajurkar

**Affiliations:** Department of Microbiology, Jawaharlal Nehru Medical College, Wardha 442004, India

## Abstract

*Background and Objective*. Antimicrobial resistance is now a major challenge to clinicians for treating patients. Hence, this short term study was undertaken to detect the incidence of multidrug-resistant (MDR), extensively drug-resistant (XDR), and pandrug-resistant (PDR) bacterial isolates in a tertiary care hospital.* Material and Methods*. The clinical samples were cultured and bacterial strains were identified in the department of microbiology. The antibiotic susceptibility profile of different bacterial isolates was studied to detect MDR, XDR, and PDR bacteria.* Results*. The antibiotic susceptibility profile of 1060 bacterial strains was studied. 393 (37.1%) bacterial strains were MDR, 146 (13.8%) strains were XDR, and no PDR was isolated. All (100%) Gram negative bacterial strains were sensitive to colistin whereas all (100%) Gram positive bacterial strains were sensitive to vancomycin.* Conclusion*. Close monitoring of MDR, XDR, or even PDR must be done by all clinical microbiology laboratories to implement effective measures to reduce the menace of antimicrobial resistance.

## 1. Introduction 

In 2011, WHO declared “combat drug resistance: no action today, no cure tomorrow.” [1]. In recent years, strains of multidrug resistant organisms have become quadrupled worldwide [2]. Presently, antimicrobial resistance (AMR) poses a major threat to patient's treatment as it leads to increased morbidity and mortality, increased hospital stay, and severe economic loss to the patient and nation [3, 4]. The clinical isolates such as* Pseudomonas aeruginosa*, Methicillin Resistant* Staphylococcus aureus* (MRSA), Enterococci especially Vancomycin Resistant Enterococci (VRE), and members of Family Enterobacteriaceae, for example,* Klebsiella pneumoniae*,* E. coli*, and* Proteus* sp., rapidly develop antibiotic resistance and spread in the hospital environment. Actually, the health care planners have declared “Health for All by the year 2000.” In the last two decades, there were so much increase of infectious diseases that the standard of public health in many parts of the world is equivalent to preantibiotic era [5]. As per standardized international terminology created by European Centre for Disease Control (ECDC) and Centre for Disease Control & Prevention (CDC), Atlanta, the multidrug-resistant (MDR), extensively drug-resistant (XDR), and pandrug-resistant (PDR) bacteria have been well defined [6]. Multidrug resistant (MDR) was defined as acquired nonsusceptibility to at least one agent in three or more antimicrobial categories. Extensively drug resistant (XDR) was defined as nonsusceptibility to at least one agent in all but two or fewer antimicrobial categories (i.e., bacterial isolates remain susceptible to only one or two antimicrobial categories). Pandrug resistant (PDR) was defined as nonsusceptibility to all agents in all antimicrobial categories.

Hence, this short term study was undertaken to detect the incidence of MDR, XDR, and PDR bacterial isolates in a tertiary care hospital of Central India.

## 2. Material and Methods

This short term cross-sectional study was conducted in the department of microbiology from 15th of April to 15th of July, 2014. The bacterial strains were isolated from different clinical samples and were identified by conventional methods [7]. The clinical specimens from indoor patient departments (IPD) only were included in the study. Antibiotic susceptibility test of bacterial strains was done by Kirby Bauer disc diffusion method [8] as per Clinical Laboratory Standard Institute (CLSI) guidelines [9].

Antibiotics used for Gram positive cocci (GPC) were penicillin, erythromycin, ciprofloxacin, tetracycline, amikacin, vancomycin, and linezolid and for Gram negative bacilli (GNB) were amikacin, ceftazidime, ceftazidime-clavulanic acid, ciprofloxacin, imipenem, and colistin, respectively. Linezolid and colistin were used as supplemental drugs. For urine sample, instead of ciprofloxacin and tetracycline, the antibiotics used were norfloxacin and nitrofurantoin, respectively. For routine Quality Control of antibiotic susceptibility test,* S. aureus* ATCC 25923,* E. coli* ATCC 25922, and* Pseudomonas aeruginosa* ATCC 27853 were used.

MDR, XDR, and PDR strains were detected as per criteria described by ECDC and CDC [6].

Methicillin Resistant* Staphylococcus aureus* (MRSA) strains were detected by* mec*A-mediated oxacillin resistance using cefoxitin disk (30 *μ*g) on Mueller Hinton (MH) agar plate inoculated with test strains as per standard disk diffusion recommendations and incubated at 33–35°C for 16–18 hours. Inhibition zone ≤21 mm with cefoxitin disk was interpreted as* mec*A positive according to CLSI guidelines [9]. Cefoxitin is used as a surrogate marker for* mec*A-mediated oxacillin resistance.* S. aureus* ATCC 43300 was used as Quality Control for* mec*A positive strains.

Extended Spectrum *β*-lactamases (ESBL) producing strains were detected by combined disk method using ceftazidime (30 *μ*g) and ceftazidime plus clavulanic acid (30 *μ*g plus 10 *μ*g) [10]. An increase in diameter of ≥5 mm with ceftazidime plus clavulanic acid as compared to ceftazidime disk alone was considered positive for ESBL detection.

## 3. Observations and Results

A total number of 880 clinical samples with bacterial growth were studied. 138 clinical samples were received from intensive care unit (ICU) and 742 clinical samples were received from wards of different clinical specialities. 698 clinical samples had single bacterial growth and 182 had mixed bacterial growth. Out of these 182 clinical samples, 172 samples had 2 bacterial isolates, 4 samples had 3 bacterial isolates, and 6 samples had 1 bacterial isolate along with* Candida albicans*. Hence, a total number of 1060 bacterial strains were studied.

**Figure 1:**
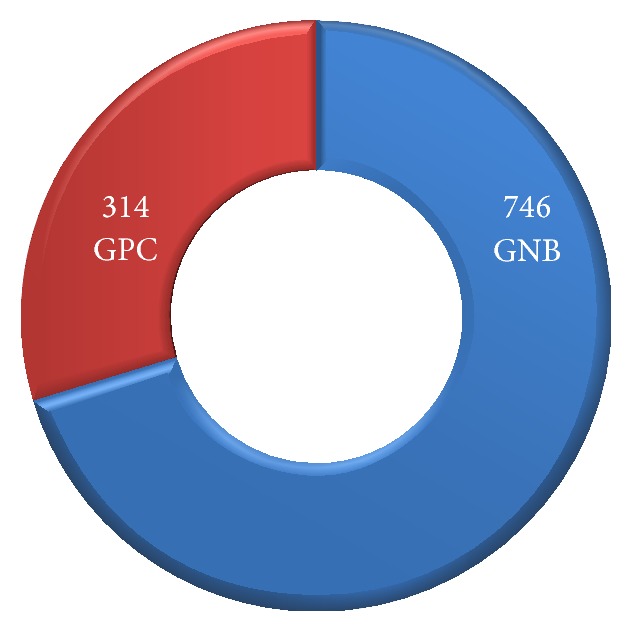
Incidence of Gram positive cocci (GPC) and Gram negative bacilli (GNB) isolated (*n* = 1060).

Figure 1 shows that 314 (29.6%) bacterial strains were Gram positive cocci (GPC) and 746 (70.4%) were Gram negative bacilli (GNB). Out of 314 GPC, 252 (80.3%) were coagulase positive staphylococci. Amongst 746 GNB, 261 (35%) were* E. coli*, followed by* Pseudomonas aeruginosa* 212 (28.4%).

During the study period, total admission in indoor patient department was 7947 and ICU admission was 1357. Hence, total patients admitted were 9304.

**Figure 2:**
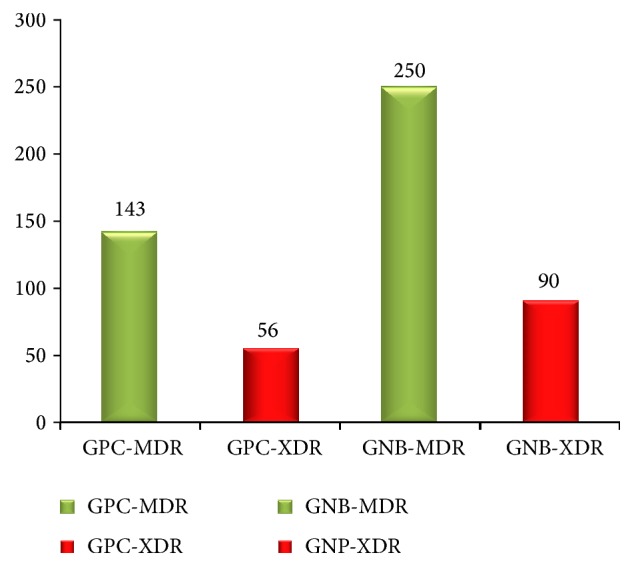
Incidence of MDR and XDR amongst total bacterial strains studied (*n* = 1060).

Figure 2 shows the incidence of MDR and XDR strains isolated. Out of total 1060 bacterial strains studied, 393 (37.1%) bacterial strains were MDR and 146 (13.8%) strains were XDR. Amongst 314 GPC strains isolated, 143 (45.5%) and 56 (17.8%) were MDR and XDR, respectively. Out of 746 GNB isolates, 250 (33.5%) strains were MDR and 90 (12.1%) were XDR. No PDR strain was detected.

Out of total 9304 patients admitted, 393 (4.2%) and 146 (1.6%) were positive for MDR and XDR strains, respectively.

**Figure 3:**
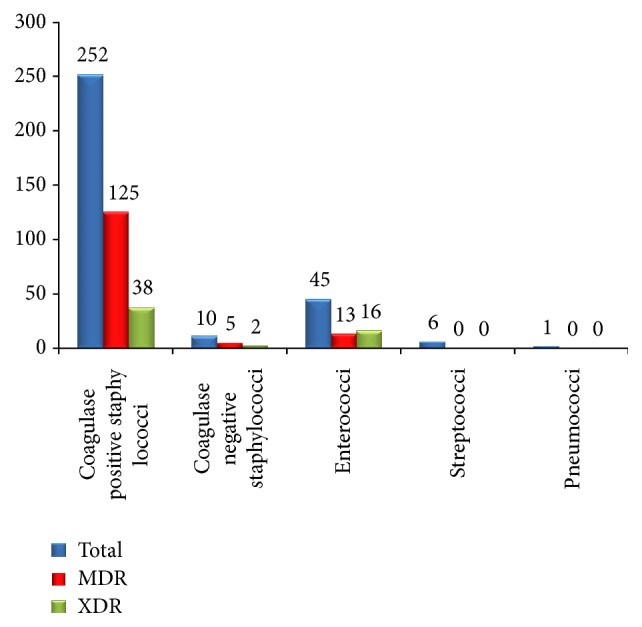
Incidence of MDR and XDR strains of each species of Gram positive cocci isolated (*n* = 314).

Figure 3 shows the incidence of MDR and XDR Gram positive cocci isolated. Out of total 252 coagulase positive staphylococci isolated, 125 (49.6%) were MDR and 38 (15.1%) were XDR. 10 coagulase negative staphylococci were isolated and 5 (50%) were MDR, whereas 2 (20%) were XDR. 79 (31.3%) coagulase positive staphylococci strains were MRSA and 2 (20%) coagulase negative staphylococci were MRCONS. Out of total 45 Enterococci isolated, 13 (28.9%) were MDR and 16 (35.6%) were XDR. No Vancomycin Intermediate* Staphylococcus aureus* (VISA), Vancomycin Resistant* Staphylococcus aureus* (VRSA), or Vancomycin Resistant Enterococci (VRE) were isolated. All* Streptococcus* species including group A, nongroup A, and pneumococcus were sensitive to penicillin. No MDR or XDR strain was isolated from* Streptococcus* sp. All (100%) Gram positive cocci were sensitive to vancomycin and linezolid.

**Figure 4:**
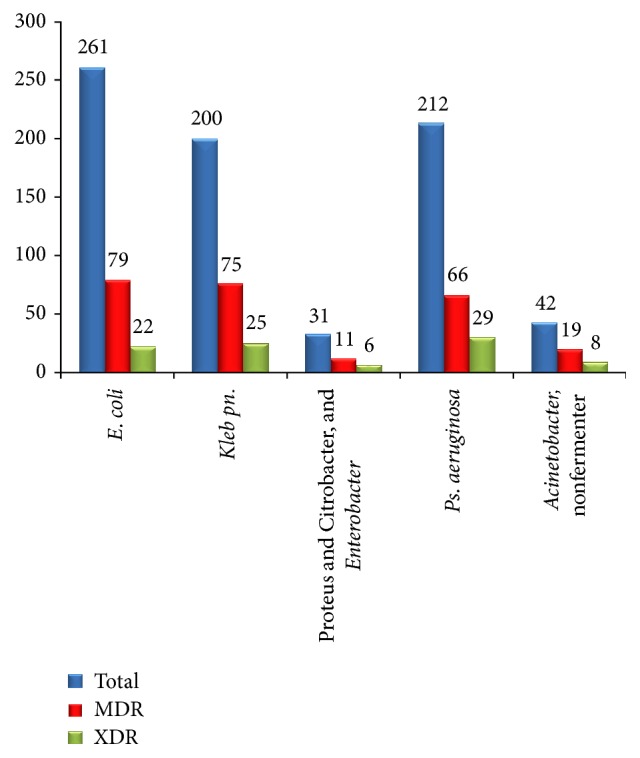
Incidence of MDR and XDR strains of each species of Gram negative bacilli isolated (*n* = 746).

Figure 4 shows incidence of MDR and XDR strains isolated from each species of Gram negative bacilli. In the present study,* E. coli* was the commonest isolate 261 (35%), followed by* Pseudomonas aeruginosa* 212 (28.4%). 79 (30.3%) and 22 (8.4%)* E. coli* strains were MDR and XDR, respectively. Out of 200* Klebsiella pneumoniae* strains isolated, 75 (37.5%) and 25 (12.5%) were detected as MDR and XDR, respectively. Out of 42* Acinetobacter* and other nonfermenter species isolated, 19 (45.2%) and 8 (19%) were MDR and XDR strains, respectively.

Amongst 250 GNB-MDR strains isolated, the commonest MDR strains were detected from* E. coli* 79/250 (31.6%), followed by* Klebsiella pneumoniae* 75/250 (30%). Similarly, out of 90 GNB-XDR strains isolated, the commonest XDR strains were detected from* Pseudomonas aeruginosa* 29/90 (32.2%), followed by* Klebsiella pneumoniae* 25/90 (27.8%).

In the present study, 137 (18.4%) ESBL producing strains were isolated. Out of 746 GNB isolated, 97 (13%) strains were imipenem (carbapenem) resistant. All (100%) Gram negative bacilli were sensitive to colistin.

**Figure 5:**
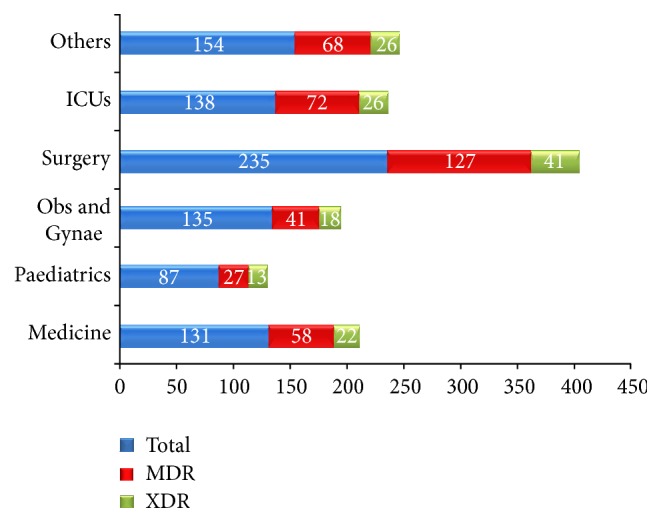
Incidence of MDR and XDR strains isolated from different clinical specialities.

Figure 5 shows the MDR and XDR strains isolated from different clinical specialities. Others include wards like dermatology, pulmonary medicine, orthopedics, and cardiovascular and thoracic surgery (CVTS). The different ICUs include Neonatal ICU (NICU), Medicine ICU (MICU), Operation Theatre ICU (OT ICU), and Paediatric ICU (PICU). Out of total 393 MDR strains detected, 127 (32.3%) (the highest number) MDR strains were isolated from surgery wards followed by 72 (18.3%) MDR strains from different ICUs. Amongst total 146 XDR strains isolated, 41 (28.1%) (the highest number) were isolated from surgery wards also. Out of 72 MDR strains detected from different ICUs, 29 (40.3%) (the highest number) MDR strains were isolated from NICU, followed by 20 (27.8%) and 18 (25%) from OT ICU, and only 5 (6.9%) from PICU. Even in the total 26 XDR strains isolated from different ICUs, 10 (38.5%) (the highest number) XDR strains were isolated from NICU.

The percentage of MDR and XDR strains isolated from different ICUs was 72/138 (52.2%) and 26/138 (18.8%), respectively, which were again much more than MDR and XDR strains isolated from wards 321/742 (43.3%) and 120/742 (16.2%), respectively.

275 patients were admitted to NICU, of whom 29 (10.5%) were positive for MDR strains and 10 (3.6%) were positive for XDR strains. Out of total 1907 patients admitted to surgery ward, 127 (6.7%) were positive for MDR strains whereas 41 (2.1%) were positive for XDR strains. In MICU, 545 patients were admitted and 20 (3.7%) were positive for MDR strains and 8 (1.5%) were positive for XDR strains.

## 4. Discussion

The clinical and financial burden to patients and health care providers for MDROs is really challenging. Barbara Soule, Joint Commission Resources Practice Leader, Infection Prevention and Control Services, has told, “Patients who are infected with MDROs often have an increased risk of prolonged illness and mortality. The cost of care for these patients can be more than double as compared to those without MDRO infection”. Since the year 2000, only 4 new classes of antibiotics have been approved by Food and Drug Administration (FDA), US, for example, linezolid, streptogramins, daptomycin, and tigecycline. The first 3 drugs are effective against MRSA and VRE. Tigecycline has also effect on Gram negative bacilli. The problem is that the bacteria are developing resistance at a much faster pace than the new drug development [11]. Regarding public health attention, MDROs are described as superbugs having very limited treatment options. For some MDROs, only 1 or 2 antibiotics can be effective with toxic side effects. In 2009, Boucher et al. have reported ESKAPE organisms as “Bad Bugs,” where E stands for* Enterococcus faecium*, S for* Staphylococcus aureus*, K for* Klebsiella pneumoniae*, A for* Acinetobacter baumannii*, P for* Pseudomonas aeruginosa*, and E for* Enterobacter* species [12]. In the year 2009 only, Peterson has reported the ESCAPE group of organism, which was the same as the above list but K was replaced by C, that is,* Clostridium difficile*, and the last E stands for Enterobacteriaceae [13].

In the present study, amongst 250 GNB-MDR strains isolated, the commonest MDR strains were detected from* E. coli* 79/250 (31.6%), followed by* Klebsiella pneumoniae* 75/250 (30%). Similarly, out of 90 GNB-XDR strains isolated, the commonest XDR strains were detected from* Pseudomonas aeruginosa* 29/90 (32.2%), followed by* Klebsiella pneumoniae* 25/90 (27.8%). Our findings correlated well with other studies. Aly and Balkhy reported that most prevalent MDRO in their study was* E. coli* followed by* Klebsiella pneumoniae* [14]. In another study, carried out in a tertiary care hospital in Riyadh, it has been reported that most frequent MDR pathogens were* Pseudomonas aeruginosa* followed by* E. coli* [15]. The percentage of MDR* E. coli* strains was more than* Klebsiella pneumoniae* and even* Pseudomonas aeruginosa* in our study probably because a total number of* E. coli* strains isolated (261) were also higher.

The slightly increased incidence of drug resistant strains observed in our study may be because our hospital is a tertiary care center in a rural setup and patients from adjoining districts and even villages are admitted for treatment. Before attending the hospital, most of the patients get different antibiotics from general practitioners or due to over-the-counter sell of antibiotics often in improper dose.

The limitation of this study is that this is a single center study for only three-month period in a tertiary care hospital in Central India. To reflect the trend of infections caused by MDR and XDR strains of bacteria in the region, a multicenter study involving all types of healthcare setups for a minimum period of one year would be needed.

There is paucity of data regarding MDROs in health care setup not only in India but also worldwide. Unless and until multidrug resistant organisms are detected and their incidence is known, the strategies for their control cannot be adopted properly in healthcare setup. Hence, detection, prevention of transmission of MDROs by following infection control practices, antimicrobial surveillance, and stewardship are need of the hour. Misuse and overuse of antibiotics, over-the-counter selling of antibiotics without prescription to common people, must be stopped by strict implementations of rules and regulations.

## 5. Conclusion

We hereby conclude that early detection and close monitoring of MDR, XDR, or even PDR bacterial strains must be started by all clinical microbiology laboratories to reduce the menace of antimicrobial resistance which is now a global problem.
